# Calcium carbonate nanoparticles stimulate cancer cell reprogramming to suppress tumor growth and invasion in an organ-on-a-chip system

**DOI:** 10.1038/s41598-021-88687-6

**Published:** 2021-04-29

**Authors:** Sandra F. Lam, Kevin W. Bishop, Rachel Mintz, Lei Fang, Samuel Achilefu

**Affiliations:** 1 Department of Radiology, Washington University School of Medicine, St. Louis, MO USA; 2 Department of Biomedical Engineering, Washington University, St. Louis, MO USA; 3 Department of Medicine, Washington University School of Medicine, St. Louis, MO USA; 4 Department of Biochemistry and Molecular Biophysics, Washington University School of Medicine, St. Louis, MO USA

**Keywords:** Nanoscale materials, Techniques and instrumentation, Imaging, Lab-on-a-chip, Materials chemistry, Cancer, Oncology, Nanoscience and technology

## Abstract

The acidic microenvironment of solid tumors induces the propagation of highly invasive and metastatic phenotypes. However, simulating these conditions in animal models present challenges that confound the effects of pH modulators on tumor progression. To recapitulate the tumor microenvironment and isolate the effect of pH on tumor viability, we developed a bifurcated microfluidic device that supports two different cell environments for direct comparison. RFP-expressing breast cancer cells (MDA-MB-231) were cultured in treatment and control chambers surrounded by fibrin, which received acid-neutralizing CaCO_3_ nanoparticles (nanoCaCO_3_) and cell culture media, respectively. Data analysis revealed that nanoCaCO_3_ buffered the pH within the normal physiological range and inhibited tumor cell proliferation compared to the untreated control *(p* < *0.05*)*.* Co-incubation of cancer cells and fibroblasts, followed by nanoCaCO_3_ treatment showed that the nanoparticles selectively inhibited the growth of the MDA-MB-231 cells and reduced cellular migration of these cells with no impact on the fibroblasts. Sustainable decrease in the intracellular pH of cancer cells treated with nanoCaCO_3_ indicates that the extracellular pH induced cellular metabolic reprogramming. These results suggest that the nanoCaCO_3_ can restrict the aggressiveness of tumor cells without affecting the growth and behavior of the surrounding stromal cells.

## Introduction

During tumor progression, cells undergo many changes to cope with the harsh environment that is created by the rapidly expanding cells. One change that occurs is the acidification of the tumor microenvironment^1–5^. This physiologic condition is driven by the upregulation of glycolysis and proton transporters that allow the cancer cells to thrive in acidic conditions^6–8^. Acidic tumor microenvironment (TME) increases the invasion, metastasis, proliferation, and drug resistance of cancer cells^1,9–14^. One approach to combat these effects is to use therapies that buffer the TME to maintain normal physiology at a pH of approximately 7.4.

Previous studies have shown that sodium bicarbonate successfully decreased tumor metastasis^15,16^. However, oral administration of the product ad libitum does not target the tumor directly but instead raises the global extracellular pH, which could induce metabolic alkalosis and potential morbidity. A variety of other approaches have been explored to modulate the acidic TME or use this phenomenon to deliver drugs via pH-responsive NPs^17,18^ and cell-penetrating peptides^19^. Among the various methods that modulate the acidic TME and enhance drug delivery^20–24^, acid-neutralizing CaCO_3_ nanoparticles (nanoCaCO_3_) have been particularly effective due to their high payload and buffering capacity, facilitating drug delivery^17,25^ and direct pH modulation of the TME^26^. NanoCaCO_3_ have few side effects, as they degrade into calcium and CO_2_ and only increase the extracellular pH to a maximum of 7.4^26^. However, deciphering the effects of nanoCaCO_3_ on tumor cells in the complex in vivo environment is challenging. Without a method to dynamically quantify the number of nanoparticles that accumulate in the tumor, the net effect of pH on inhibiting the acidic extracellular pH in tumors remains unclear. This situation can cause significant variability in the results obtained and requires extensive ex vivo analysis to identify how nanoCaCO_3_ treatment affected tumor migration.

In this study, we determined the effect of pH changes on tumor survival and migration by simulating in vivo flow parameters in a controlled environment using microfluidic devices. Microfluidic devices have the ability to grow multiple cells in a 3D environment and offer precise control of fluid flow^27–30^. Using a transparent polymer, polydimethylsiloxane (PDMS), for device fabrication permits real-time fluorescence microscopy of the cell growth. A bifurcate design allows half of the tissue chambers to receive nanoCaCO_3_, while the other half receives cell culture medium. The design enabled us to directly compare the growth of tumor cells treated with small-sized nanoCaCO_3_ (< 120 nm) to those without treatment on the same device. We demonstrate that not only did nanoCaCO_3_ increase the extracellular pH asymptotically to 7.4, but they also inhibited tumor growth and migration.

## Materials and methods

### Cell culture

RFP expressing MDA-MB-231 human metastatic breast cancer cells were purchased from Cell Biolabs (San Diego, CA), and skin fibroblast cells were purchased from the Coriell Institute (Camden, New Jersey). MDA-MB-231 cells served as an appropriate model for our study because of their sensitivity to acidic TME^31^. All cells were grown in Dulbecco’s Modified Eagle Medium (DMEM, ThermoFisher, Waltham, MA) containing 10% fetal bovine serum (FBS, Sigma-Aldrich, St. Louis, MO), 1% L-glutamine (ThermoFisher), and 1% penicillin streptomycin (ThermoFisher). They were cultured in a humidified incubator at 37 °C and 5% CO_2_.

### Microfluidic device design and simulations

**Figure 1:**
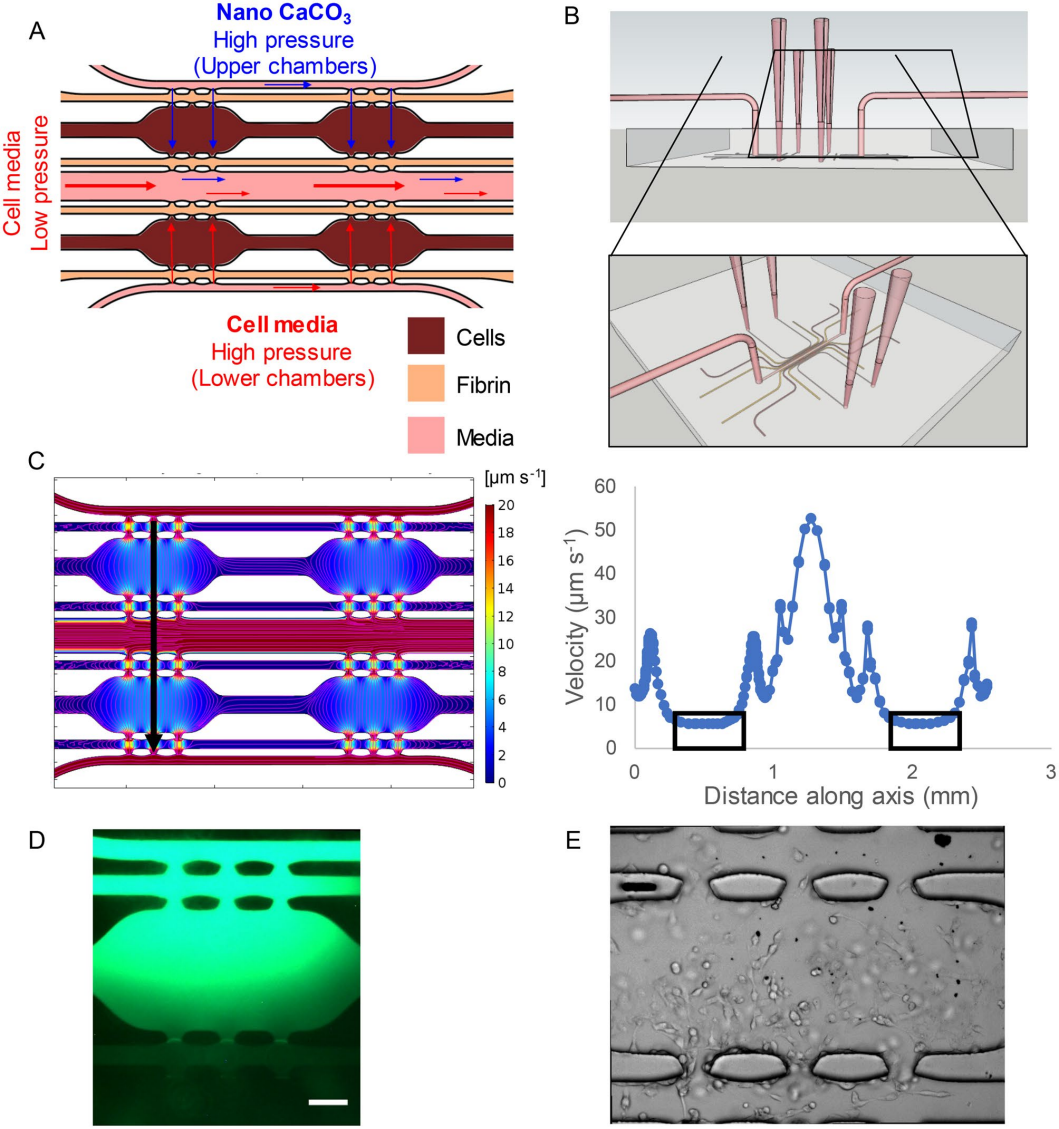
Device design and setup. (**A**) Design of the microfluidic device. Cells in fibrin are loaded into the brown chambers. Plain fibrin is loaded in the adjacent chambers to quantify cellular migration. Media is flown through the pink channels to feed the tissue. The flow patterns of the device are illustrated by the arrows. The two upper chambers will receive media with CaCO_3_ nanoparticles (blue arrows), while the two lower chambers will receive plain media (red arrows). The central chamber is connected to a microfluidic pump to serve as a “waste” stream so the upper and lower chambers can maintain independence from each other. (**B**) Experimental setup of the microfluidic device. Pipette tips are used to feed the tissue chambers on the outside of the device, while the middle media channel is connected to a microfluidic pump via tubing. (**C**) Fluid velocity (µm s^−1^) distributions are shown in the surface map (left). The streamlines of interstitial flow are illustrated with pink lines. Quantification of velocities along the black arrow are shown in the graph to the right. The boxed regions in the graph represent the fluid velocities within the tissue chambers. (**D**) FITC-dextran flow through the top media channel to show that the fluorescent signal is attenuated towards the lower chambers. This confirms that there is no crosstalk between the upper and lower chambers. Scale bar = 200 µm. (**E**) Visual confirmation of the presence of CaCO_3_ nanoparticles. The black punctate marks in the tissue chamber and media lines serve as a visual representation that the CaCO_3_ nanoparticles are reaching the tissue chamber through the media lines.

We developed a microfluidic device that can incorporate the experimental and control conditions on the same chip. Two tissue chambers (0.061 mm^3^) were located near the upper portion of the device (experimental), and two were located near the lower portion of the device (control). Smaller chambers of plain fibrin (0.015 mm^3^) were placed adjacent to the tissue chambers to measure cellular migration. Lastly, media channels were placed on the outside of fibrin chambers. By manipulating the hydrostatic pressure difference between media channels, we were able to control fluid velocities to mimic physiological values. To ensure proper separation between the experimental and control tissue chambers, a larger “waste” channel was placed between the two compartments to inhibit contamination between chambers (Fig. 1A). Additionally, flow through this channel was constantly maintained via connection to a microfluidic syringe pump.

We then used AutoCAD to construct a computer-aided design of the device. Standard photolithography methods were used to create a silicon mold of the microfluidic devices^32^. Briefly, SU-8 2050 (MicroChem, Newton, MA) was spun on to a silicon wafer to a height of 100 μm. Using the printed pattern, the design was crosslinked into the photoresist with ultraviolet exposure. After developing the pattern out of the mold, it was silanized before pouring polydimethylsiloxane (PDMS, Dow Corning, Elizabethtown, KY) on top of it. The PDMS mixture was prepared from a 10:1 (w/w) polymer to curing agent solution, which was mixed thoroughly. After degassing the PDMS, it was cured overnight in a 65 °C oven. The PDMS was then peeled off, and the inlet and outlets of the device were punched with a 16-gauge needle. To bond the PDMS device to a glass slide, both pieces were cleaned and placed under plasma treatment for 1 min at 250 mTorr. After sealing the pieces of the device to the glass slide, the material was placed in a 120 °C oven for at least 15 min. The device was sterilized under ultraviolet light for at least 8 h before use.

We used COMSOL Multiphysics 5.4 (Burlington, MA) to simulate interstitial flow within the microfluidic devices. A 2D steady-state solution of incompressible Navier–Stokes equations was solved to calculate flow throughout the device using the *Porous Media Flow* module. A no slip boundary condition was used for all the walls, and the properties of the device have been previously published^27,33^.

### NanoCaCO_3_ fabrication and size characterization

NanoCaCO_3_ were synthesized using a gas diffusion method^34^. Briefly, a solution of CaCl_2_ hexahydrate (1 g; 4.6 mmol; Sigma–Aldrich) in 200 mL of anhydrous ethanol was added to a 400 mL beaker, which was covered with Parafilm in a desiccator containing Drierite. To allow gas exchange, the Parafilm cover was punctured with small holes. The beaker was surrounded by 16 glass vials (20 mL) filled with dry ammonium bicarbonate (typically 20 g per vial). Seeding and nanoparticle formation was allowed to occur over 5 days in a vacuum. The solid nanoparticles were obtained by centrifuging the suspension in ethanol at 6800 g for 10 min in 1 mL Eppendorf tubes, air-dried over 24 h, and stored at 4 °C. The materials were reconstituted in DMEM before use.

Transmission electron microscopy (TEM) images of the nanoCaCO_3_ were captured using a FEI Tecnai Spirit Transmission Electron Microscope (FEI, Hillsboro, OR, USA) operating at an acceleration voltage of 200 kV, as described previously^34^. TEM grids coated with a layer of Formvar were used throughout these studies. Dynamic light scattering measurements of the nanoparticles in ethanol, distilled water, phosphate buffered saline (PBS), or serum were acquired with a Malvern Zetasizer Nano ZS (Malvern Instruments Ltd., Malvern, UK) instrument equipped with a 633 nm laser. Three measurements were conducted for each sample with at least 10 runs, each run lasting 10 s. All sizes reported were based on the intensity average.

### Microfluidic experimental design and setup

For each experiment, fibrin was used to create the extracellular matrix for the TME. Fibrinogen (Sigma-Aldrich, St. Louis, MO) and thrombin (Sigma-Aldrich) were prepared in PBS (Gibco) for a final concentration of 16 mg mL^−1^ and 50 U mL^−1^ respectively. MDA-MB-231 cells were trypsinized and resuspended in the fibrinogen solution at a concentration of 3 × 10^6^ cells mL^−1^. The thrombin was then added to this mixture to initiate the polymerization process. Before the solution completely polymerized, it was pipetted into the tissue chambers of the device. Immediately after loading the cells into the device, plain fibrin was loaded to the adjacent chambers using the same method. The device was then placed in a 37 °C incubator for 30 min before media was added to the media channels Tygon tubing (Saint-Gobain, Valley Forge, PA) was connected to the inlets and outlets of the waste stream channel and attached to a syringe pump (Fig. 1B). A syringe containing media was flowed constantly for a rate of 10 nL s^−1^ throughout the experiments. After the device was placed in the incubator overnight, the media in the top channel was replaced with the DMEM media containing nanoCaCO_3_ at selected concentrations of ≤ 0.8 mg mL^−1^. The media was changed every 2 days for the 6-day experiment. For the experiment containing cancer and fibroblast cells, the cells were loaded at a concentration of 1.71 × 10^6^ cells mL^−1^ and 4.29 × 10^6^ cells mL^−1^, respectively.

### Imaging and tumor progression analysis

Both fluorescent and brightfield images were acquired at the start and end of each experiment using an EVOS FL Cell Imaging System (ThermoFisher). In the experiments containing fibroblasts, a live stain was used at the end of the experiment. Briefly, Calcein AM was used to determine cell viability and label the live cells with green fluorescence. First, the cells were washed with Hanks’ Balanced Salt Solution (HBSS, Gibco) for an hour before incubating the cells in the dye for at least 2 h. All images were cropped to contain either the top, center, or bottom chambers. MDA-MB-231 tumor area was measured with ImageJ by calculating the red fluorescent area. The fibroblast cells remained nonfluorescent to ensure their properties were not altered, and their area was traced manually during image analysis to calculate the fibroblast area on the initial day of loading. At the end of the experiment, fibroblast area was determined by subtracting the red fluorescent area (MDA-MB-231 cell area) from the green fluorescent area (total viable cellular area). The parameters for growth and migration were calculated using the following equations:

$$Growth\,ratio= \frac{Cellular\,area\,on\,final\,day}{Cellular\,area\,on\,initial\,day}$$

$$Migration=\frac{Total\,cellular\,area\,in\,the\,top\,or\,boottom\,chamber}{Total\,cellular\,area\,in\,the\,central\,chamber}$$

### pH measurements

To estimate how nanoCaCO_3_ were affecting the tumor microenvironment, pH measurements were made using 24 well plates. First, MDA-MB-231 cells were seeded in the wells at a concentration of 3.0 × 10^6^ cells mL^−1^. The next day, the media was replaced with either plain media (control) or media containing 0.8 mg mL^-1^ nanoCaCO_3_ (experimental). After 3 more days, the media was collected, and a pH probe (AB15 Basic, ThermoFisher) was used to measure the pH of the media in the wells.

Additionally, the intracellular pH was determined using a pHrodo Green AM Intracellular pH Indicator (ThermoFisher) following the manufacture’s protocol. With this dye, higher fluorescent intensities indicate a lower pH. Therefore, to correlate fluorescent intensities with pH values, a pH Calibration Buffer Kit (ThermoFisher) was used.

### Statistical analysis

To test the effectiveness of nanoCaCO_3_ on tumor growth inhibition, a Mann–Whitney test was used to compare the control with the experimental data sets. GraphPad Prism was used for these calculations, and the criterion for statistical significance was *p* < 0.05. All results are presented as mean ± standard deviation.

## Results

### Microfluidic device characterization

We developed a bifurcated microfluidic device that allowed two cell environments to be compared side by side (Fig. 1). The device was designed to control interstitial flow rates depending on the drop in pressures along the tissue chambers. A low-pressure waste stream flowed between the control and treatment sides. Media diffused through each chamber from high pressure to low pressure and was cleared by the central waste stream. We incorporated 6 pores that connect the tissue chambers to fibrin chambers and media channels, which were specifically designed to be capillary burst valves to maintain the cells in each compartment during the cellular loading process. Fluid flow direction was determined by the difference in hydrostatic pressure head between the top and bottom channels. The flow was designed to incorporate velocities between 1–10 µm s^−1^ to replicate physiological interstitial fluid flow (Fig. 1C). Fluid velocities were quantified along the black arrow and plotted as velocity vs. distance (Fig. 1C). The velocities in the tissue chamber are represented by the black boxes. The tissue chambers have interstitial flow rates in the physiological range of 1–10 µm/s. This event was also observed in the waste channel stream, which was pumped at a fast flow (up to 55 µm s^−1^) rate to prevent cross-communication between the experimental and control conditions. To further confirm this process, fluorescein isothiocyanate-dextran (FITC-dextran) was flown through the top media channel for a day and then imaged. No fluorescent signal in the bottom chambers was observed (Fig. 1D), indicating that the experimental and control conditions can co-exist on the same device without cross-contamination.

### NanoCaCO_3_ synthesis and characterization

**Figure 2:**
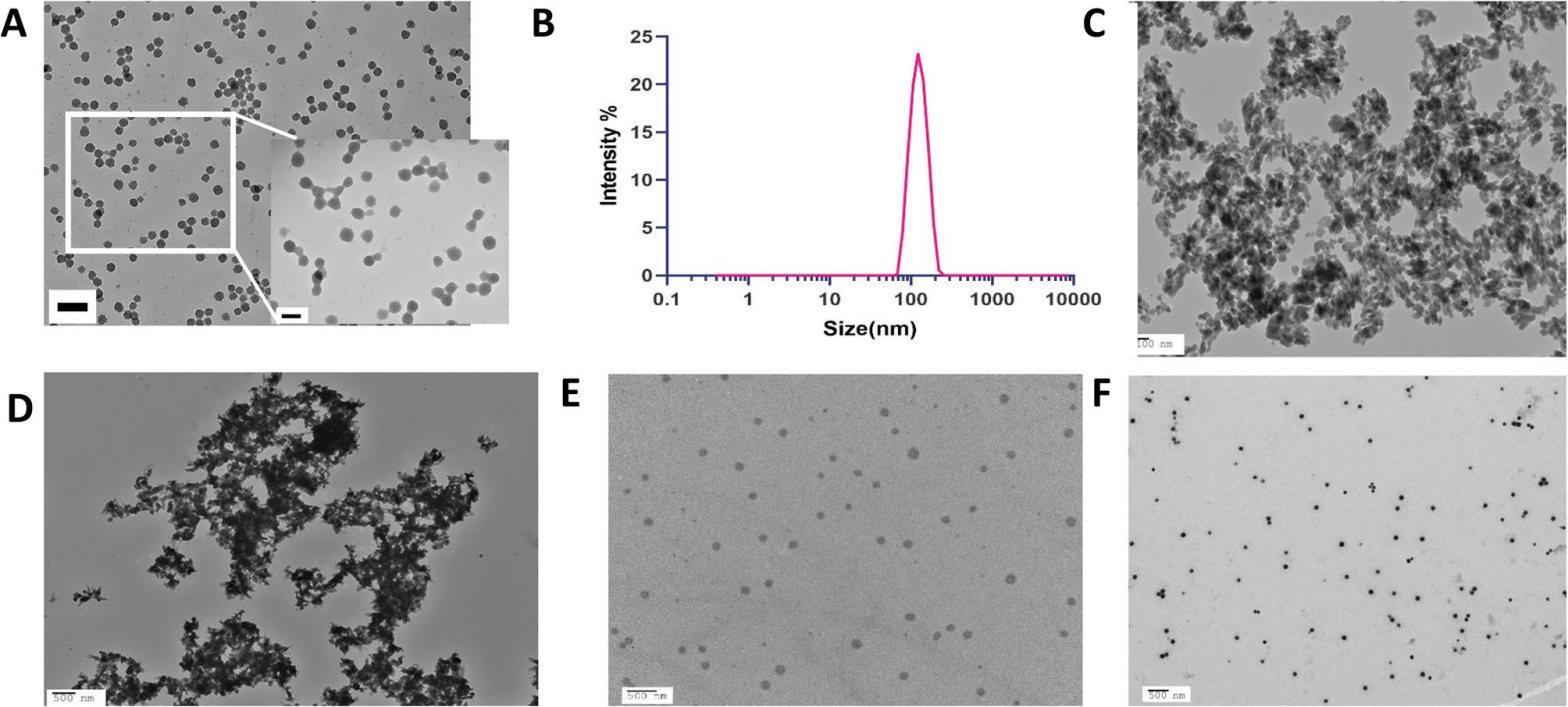
CaCO_3_ nanoparticle characterization. (**A**) Transmission electron microscope image of nanoCaCO_3_ dispersed in ethanol before drying the nanoparticles shows spherical particles with the size of 78.6 ± 7.6 nm. Scale bar = 200 nm (main panel); 100 nm (inset). (**B**) NanoCaCO_3_ were dispersed in ethanol for dynamic light scattering measurement, showing an average intensity-based size of 120.0 nm ± 29.3 nm. Average number of particles-based size measurement is 101.8 ± 24.7 nm (not shown). (**C**–**F**) TEM images of nanoCaCO_3_ after dispersion in distilled water (**C**), PBS (**D**), serum (**E**), and DMEM (**F**); scale bars = 100 nm for (**C**) and 500 nm for (**D**–**F**).

NanoCaCO_3_ were prepared by the method reported previously^34^. The transition of the solution from clear to white and cloudy indicates the nucleation and formation of the nanoparticles. Depending on the particle size desired, this reaction can be stopped at different time points. We obtained optimal yield and stable product by reacting 1 g CaCl_2_ in 200 mL ethanol with ammonium carbonate, a carbonate source over 5 days. The size and morphology of the synthesized nanoCaCO_3_ were determined by TEM and dynamic light scattering (DLS) (Fig. 2). A representative TEM micrograph revealed that the nanoCaCO_3_ were primarily spherical, with an average size of 78.6 ± 7.6 nm (Fig. 2A). The hydrodynamic diameter (Dh) size of 120.0 nm ± 29.3 nm from DLS (Fig. 2B) was larger than the TEM mean size, probably due to a combination of solvation effect and aggregation^35^. Therefore, we investigated the stability of nanoCaCO_3_ in distilled water, PBS, and serum. Unlike the TEM images for the nanoCaCO_3_ in ethanol, we found that suspensions of the nanoparticles in water or PBS formed large aggregates (Fig. 2C,D), suggesting that the less than 30% DLS size increase in ethanol is consistent with previous reports^35^. In contrast, resuspension of the nanoparticles in serum retained a high level of monodispersity (Fig. 2E). For this reason, we dispersed the nanoCaCO3 in media (DMEM) containing 10% FBS throughout this study (Fig. 2F).

### Dose–response of MDA-MB-231 cells to nanoCaCO_3_

**Figure 3:**
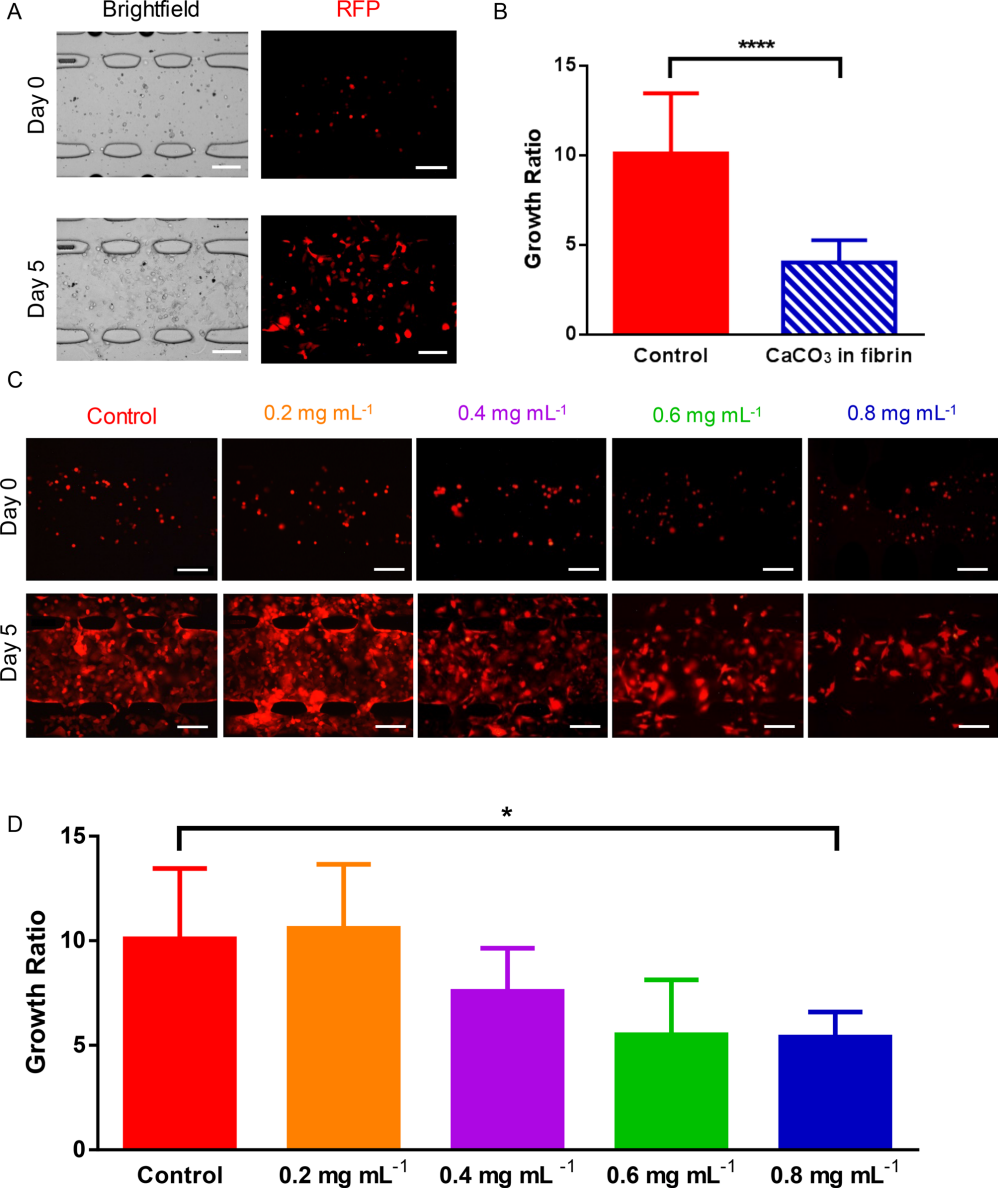
Nano CaCO_3_ effects on MDA-MB-231 cell growth. (**A**) Representative images of the MDA-MB-231 cells loaded in fibrin gels. Images of the start (top) and end (bottom) of the experiment with fibrin gels containing 1.6 mg mL^−1^ concentration of CaCO_3_ nanoparticles is shown. Scale bar = 200 µm. (**B**) Embedding CaCO_3_ nanoparticles inside fibrin. After embedding CaCO_3_ nanoparticles inside our fibrin gel, the quantification of tumor growth showed significantly more growth under the control conditions. **** p < 0.0001. (**C**) Representative images of MDA-MB-231 cells expressing RFP grown in varying concentrations of CaCO_3_ nanoparticles. Images of the tissue chambers right after loading are shown in the top row, and images at the end of the experiment are shown in the bottom row. Scale bar = 200 µm. (**D**) Quantification of the effects of CaCO_3_ nanoparticles at varying concentrations. At 0.8 mg mL^−1^, tumor growth was significantly inhibited compared to the control condition. * p < 0.05.

MDA-MB-231 cells were loaded in the central tissue chamber and plain fibrin in the adjacent chambers (Fig. 1A). The control (bottom) and treatment (top) chambers received only media and nanoCaCO_3_, respectively. Although both chambers were placed on the same device, the flow of media through the waste stream ensures that the two conditions do not come in contact with each other. Treatment chambers received 0.2, 0.4, 0.6, and 0.8 mg mL^−1^ solution of nanoCaCO_3_, and imaging was performed over the time course of the experiment. Brightfield images of black punctate specs inside the tissue chamber provided visual evidence that the nanoCaCO_3_ entered the tissue chamber (Fig. 1E). Migration of nanoCaCO_3_ embedded inside the fibrin at a concentration of 1.6 mg mL^−1^ provided additional visual evidence that the particles reached the cancer cells (Fig. 3A).

A comparison of the cell proliferation rate between control and chamber treated with 0.2 mg mL^−1^ at day 5 did not show any statistical difference, with the control group exhibiting an average of 10.09 ± 3.37 growth ratio (Fig. 3). In contrast, cells treated with nanoCaCO_3_ concentrations > 0.2 mg mL^−1^ displayed a concentration-dependent inhibition of tumor growth (Fig. 3D). The lowest tumor growth ratio of 5.40 ± 1.20 was observed for the 0.8 mg mL^−1^ treatment group, which was significantly lower than the control group (*p* < 0.05) at day 5. Furthermore, the quantification of the growth ratios shows that the MDA-MB-231 cells grown in the fibrin embedded with nanoCaCO_3_ had significantly lower growth than the control cells (Fig. 3B). With the goal of maximizing tumor inhibition, we used 0.8 mg mL^−1^ of nanoCaCO_3_ in all subsequent experiments.

### pH quantifications

**Figure 4:**
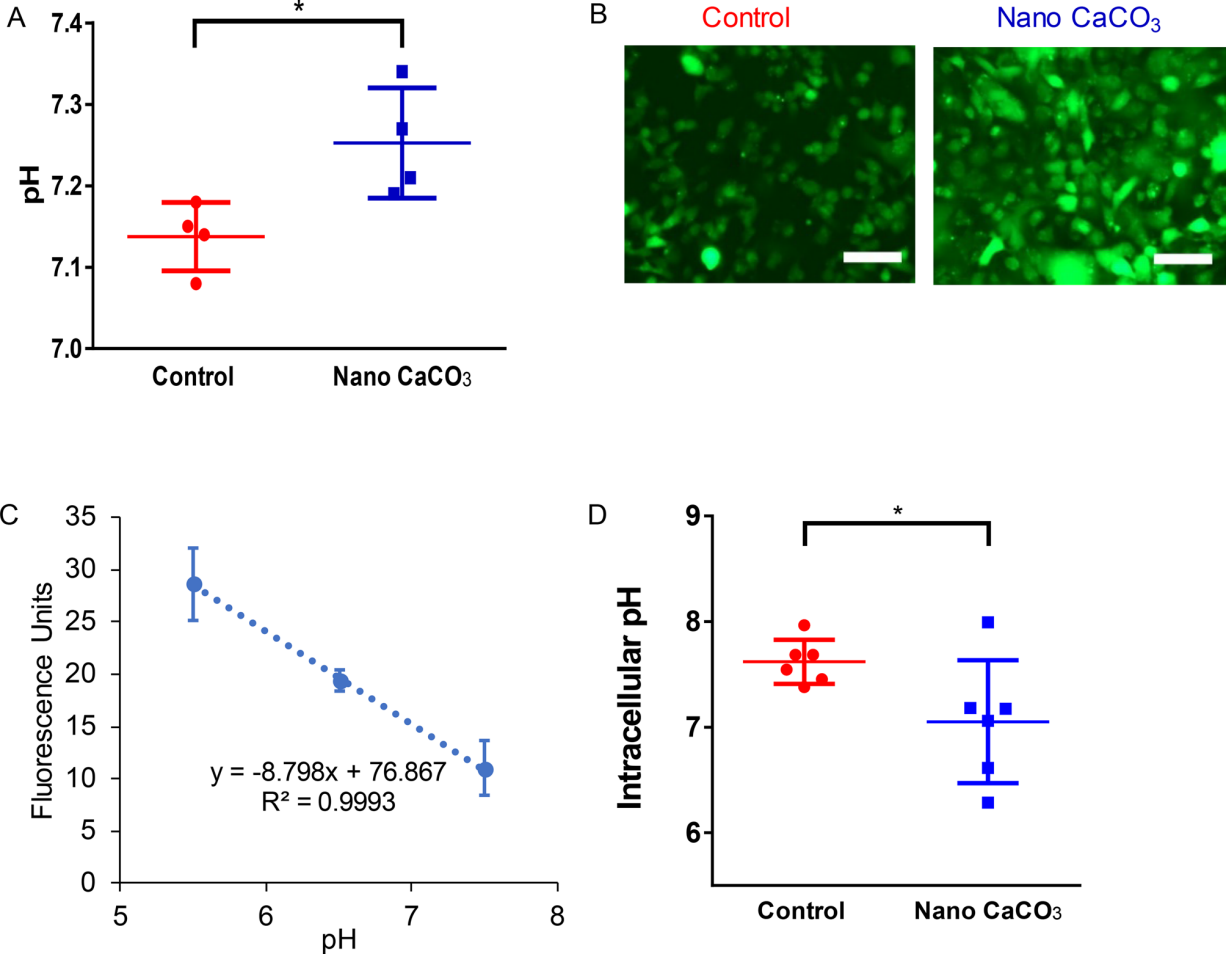
Nano CaCO_3_ effects on pH. (**A**) After the cells were seeded in 24 well plates and received media with or without CaCO_3_ nanoparticles, the media was collected and a pH probe was used to make measurements. The control group (red) had significantly lower pH than the experimental group (blue). (**B**) A pHrodo Green AM Intracellular pH Indicator dye was used compare the intracellular pH of the tumor cells with or without CaCO_3_ nanoparticles. (**C**) Higher fluorescent intensity indicates lower intracellular pH, and a Calibration Buffer Kit was used to correlate fluorescent intensity with intracellular pH. (**D**) The average intracellular pH of the MDA-MB-231 cells without nanoparticles was significantly higher than those grown with nanoparticles indicating a more alkaline intracellular pH. Scale bar = 100 µm, * p < 0.05.

Acidic TME occurs as cancer cells proliferate. To simulate this in vivo process, we cultured MDA-MB-231 cells in 24 well plates and allowed the cells to grow for 3 days with or without nanoCaCO_3_. Subsequent measurement of the media pH showed that the control group was significantly more acidic (average pH of 7.14 ± 0.04) than cells treated with nanoCaCO_3_ (average pH of 7.25 ± 0.07), reflecting the buffering effects of the nanoparticles (Fig. 4A). Changing the extracellular pH of cancer cells is expected to induce a response intracellularly^36^. Using an intracellular pH sensor (pHrodo Green AM) that increases its fluorescence intensity with pH increase^37^, we found that MDA-MB-231 cells exposed to nanoCaCO_3_ had significantly higher fluorescence intensity compared to the control group (Fig. 4B). We used a Calibration Buffer Kit to create a linear relationship between fluorescence intensity and intracellular pH (Fig. 4C). Correlating the intensity to a specific pH using this plot, we were able to determine the intracellular pH of 7.6 ± 0.09 and 7.05 ± 0.23 for the untreated and treated cells, respectively (Fig. 4D).

### NanoCaCO_3_ do not inhibit fibroblast growth, but do affect tumor migration

**Figure 5:**
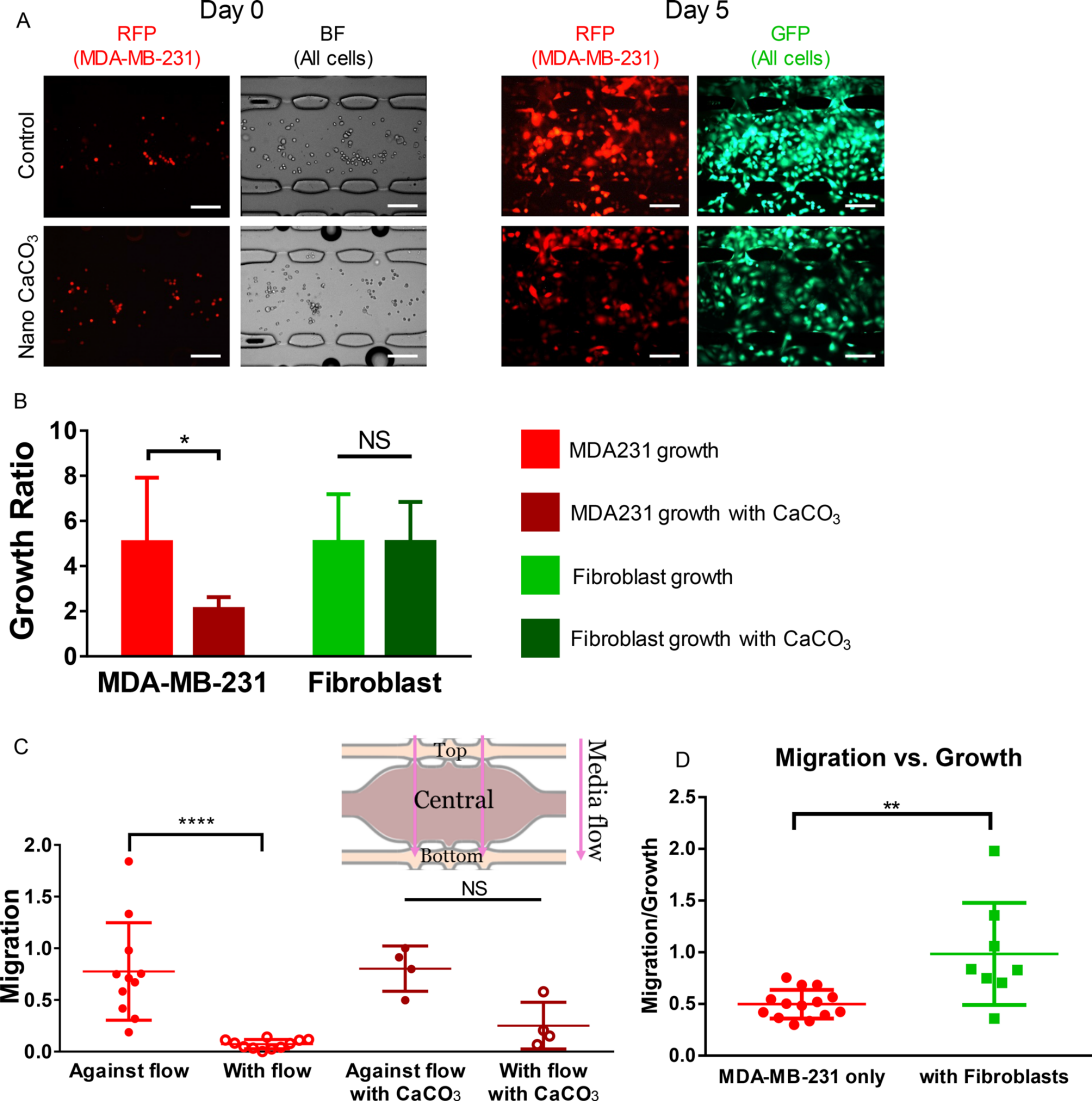
CaCO_3_ nanoparticle effects on varying tissue types. (**A**) Fluorescent and brightfield microscopy images of the control chambers (top row) and experimental chambers (bottom row) fed with CaCO_3_ nanoparticles. The left two columns include images taken right after loading of MDA-MB-231 expressing RFP and brightfield images of both cancer and fibroblast cells. The right two columns are images of the MDA-MB-231 cells at the end of the experiment as well as a live stain shown in GFP. Scale bar = 200 µm. (**B**) Quantification of the growth of MDA-MB-231 and fibroblast cells with and without CaCO_3_ nanoparticles. The data shows that there was significant inhibition of tumor growth when the nanoparticles were introduced. On the other hand, there was no significant difference between the fibroblasts that were grown with or without nanoparticles. *, p < 0.05; NS, not significant. (**C**) Quantification of cellular migration with and against the flow of media. Without the presence of nanoparticles, the MDA-MB-231 cells migrate against flow, suggesting more aggressive behavior. In the presence of nanoparticles, there was not a significant difference between migration with or against flow. This suggest that CaCO_3_ nanoparticles can inhibit the invasive behavior of MDA-MB-231 cells without the nanoparticles. ****, p < 0.0001. (**D**) MDA-MB-231 cells in the presence of fibroblasts are more migratory than without fibroblasts. To compare migratory vs growth of the MDA-MB-231 cells, we calculated a parameter that divided the cell area in the top and bottom chambers (migration) by the cell area in the middle chamber (growth). Using this parameter, we can see that the MDA-MB-231 cells growth with fibroblasts (red) are significantly more migratory than the devices with only tumor cells (green).

Cancer-associated fibroblasts (CAFs) constitute the major component of breast cancer stroma^38,39^. Through the fibroblast-tumor cell signaling axis, cancer cells can migrate, escape treatment and develop resistance. To simulate this in vivo condition, we co-incubated fibroblasts with MDA-MB-231 cells in the tissue chamber and measured the growth ratio of each cell type (Fig. 5A). Analysis of the growth pattern showed that the control group had a growth ratio of 5.06 ± 2.85 for the MDA-MB-231 cells and 5.07 ± 2.12 for the fibroblast cells (Fig. 5B). In cells treated with 0.8 mg mL^−1^ nanoCaCO_3_, the nanoparticles significantly inhibited the cancer growth ratio (2.10 ± 0.53) but not the fibroblast growth ratio (5.07 ± 1.77). These results demonstrated that nanoCaCO_3_ can selectively inhibit tumor growth without harming surrounding non-tumor host cells such as fibroblasts. However, given the dual role of CAFs in the TME^40^, the full impact of this selective inhibition is unknown at this time.

An essential function of cancer-associated fibroblasts is to support tumor migration^41^. Co-incubation of fibroblasts with MDA-MB-231 cells showed that the cancer cells were more migratory than in the absence of fibroblasts (Fig. 5C). Additionally, the MDA-MB-231 cells with fibroblasts had statistically more migration against the flow of media (top migration chamber) than with the flow of media (bottom migration chamber). The migration parameter was 0.78 ± 0.47 against flow and 0.07 ± 0.45 with flow. However, with the addition of nanoCaCO_3_, this migration pattern was diminished and not statistically significant, as indicated with the migration parameter values of 0.80 ± 0.22 and 0.25 ± 0.23 against and with flow respectively (Fig. 5D). The moderate increase of cancer cell migration with flow upon treatment with nanoCaCO3 suggests that these nanoparticles could mobilize cancer cells^42^, making them more susceptible to chemotherapy when used as combination therapy.

## Discussion

Acidic TME is one of the hallmarks of solid tumors^1^. Previously considered a side product of the Warburg effect, new evidence supports the view that cancer cells intentionally harness acidic conditions to activate tissue degrading enzymes and alter the metabolism of stromal cells^3^. Tumor acidity is associated with invasive and metastatic behavior, which leads to higher morbidity rates^43,44^. Previous studies have used this phenomenon to activate or release drugs selectively in the acidic TME^45^. An interesting development is the potential use of acid-neutralizing products to inhibit tumor growth^45^. Studies using sodium carbonate infusion clearly demonstrated that the neutralizing effect can inhibit tumor growth^44^. Unfortunately, clinical translatability of this approach is not realistic due to the potential induction of alkalosis. Recent studies have attempted to overcome this challenge by using nanoparticles to buffer the TME, thereby preventing an increase in acidity^46,47^. Regardless of the method, the complex in vivo environment complicates the delivery and prolonged retention of the buffering agents in the TME.

Organ-on-a-chip technologies present a unique opportunity to culture cells in a 3D microenvironment that is more representative of human physiology than the traditional 2D cell culture. In addition, microfluidic devices can provide precise control over factors such as chemical gradients and fluid flow velocities. Thus, this platform can be used to simulate critical factors of human microenvironments, such as interstitial fluid flow. Other studies have used microfluidic devices to study MDA-MB-231 cells^48^. However separate conditions are often implemented on separate devices. In this study, we developed microfluidic devices that have four separate cell culture chambers with the ability to receive both media and agents for diverse treatment conditions. By supporting multiple conditions on the same device, our system minimizes variability by maintaining identical temperature or gas (O_2_ and CO_2_) levels throughout the device while seamlessly changing the media simultaneously to optimize test conditions. The versatility of our system allows us to incorporate extracellular matrix proteins such as fibrin and co-incubate stromal cells such as fibroblasts with tumor cells to simulate the in vivo TME. Employing microfluidic techniques, we have shown that persistent buffering of the TME with nanoCaCO_3_ inhibited tumor growth and migration. By connecting a waste stream to a fluidic pump, both the treatment (upper chambers) and control (lower chambers) conditions were implemented on the same device for direct comparison.

Quantification of the TME acidity revealed that cells treated with nanoCaCO_3_ in well plates maintained an average pH of 7.25 ± 0.07, which is significantly higher than the control cell population that had an average pH of 7.14 ± 0.04. Although the expected pH of 7.4 was not attained, the differential buffering capacity of nanoCaCO_3_ compared to the untreated control was significant under static conditions. Given the small size of the cell population, the actual extracellular pH may be higher if measured adjacent to the secreting membrane regions. Under dynamic conditions, we imagine the buffering capabilities of the nanoCaCoO_3_ would bring the system to a pH closer to the expected 7.4 value.

Previous studies have shown that cancer cells undergo significant reprogramming in response to changes in the extracellular pH^49^. To test if nanoCaCO_3_ altered cancer cell metabolism, we measured the intracellular pH response to extracellular pH perturbation. The control group had a more alkaline intracellular pH (7.60 ± 0.09) than the treated group (7.05 ± 0.23). This result is consistent with the findings that during tumor progression, the intracellular pH becomes more alkaline as the extracellular pH decreases^50–53^. Our results suggest that nanoCaCO_3_ induced cell reprogramming in response to the change in the microenvironment. The significant increase in intracellular acidity points to a mechanism where nanoCaCO_3_ alters the efflux rate of protons in the cells. If the observed reduction in cancer cell proliferation in the presence of nanoCaCO_3_ is caused by an increase in the intracellular proton concentration, coupling nanoCaCO_3_ therapy with drugs that are effective under acidic conditions will usher a new combination therapeutic strategy for cancer.

Another interesting observation from this study is the effect of fibroblasts on the behavior of MDA-MB-231 cells. Co-incubation of the tumor cells with fibroblasts increased the migratory characteristic of the cancer cells, indicating the enhancement of a more invasive tumor phenotype. This tendency decreased in the presence of nanoCaCO_3_. Furthermore, with the fibroblasts present, the tumor cells had a penchant for migrating against the flow of media. This observation recapitulates in vivo conditions where cancer cells adopt the spindle shape of fibroblasts to propel their metastatic tendencies against flow gradients^54^. Together, our results suggest that tumor cells grown in the presence of fibroblasts exhibit more aggressive behavior than without fibroblasts. Lastly, nanoCaCO_3_ inhibited the migratory effects of MDA-MB-231 cells in the presence of fibroblasts. While the molecular mechanisms of these mechanochemical events need further studies, the pronounced effects of nanoCaCO3 on cancer cells compared to CAFs could be attributed to the inverse role these cells play in the TME. For example, CAFs are known to secrete proteins in the TME that reabsorb the lactate cancer cells produce^55^, suggesting that nanoCaCO3 will have minimal effect on the non-lactic acid producing cells. Our work adds to the body of evidence supporting the inclusion of drugs that can sustainably buffer the extracellular pH of solid tumors in combinational therapies. Extension of this promising finding to other cancer cell types will determine the broad applicability of the phenomenon in diverse cancer treatment.

With the ability to grow various cell types in the same device, we have shown that nanoCaCO_3_ do not affect fibroblast growth. While the nanoparticles significantly decreased the tumor growth, there was no significant change in fibroblast proliferation. An interplay between cancer cell killing therapies and those that can protect stromal cells requires knowledge of combination therapies that will achieve maximum effect. Although additional studies are warranted, our result points to a paradigm where nanoCaCO_3_ could sensitize cancer cells to standard of care therapies without damaging stromal cells.

## Conclusions

The microfluidic device provides a platform to expand drug testing and examine the effects of cancer therapies on multiple organ types. Using microfluidic devices, we can precisely control the cellular microenvironment. This approach allowed us to recapitulate some in vivo conditions under well-controlled conditions. Our results reveal the multidimensional therapeutic effects that nanoCaCO_3_ can exert on cancer cells. By buffering the extracellular pH of tumors above 7.2, nanoCaCO_3_ were able to alter the trajectory of cancer cells by increasing the intracellular acidity, inhibiting the migratory tendencies, and preventing the proliferation of more invasive phenotypes of these cells without harming non-tumor cells. Each of these effects presents an opportunity to leverage the vulnerability of cancer cells in the presence of nanoCaCO_3_ to design combination therapies and potentiate the therapeutic effect of drugs.

## Data Availability

All data used in this study will be shared upon request.
